# Food and nutrition security definitions, constructs, frameworks, measurements, and applications: global lessons

**DOI:** 10.3389/fpubh.2024.1340149

**Published:** 2024-03-14

**Authors:** Rafael Pérez-Escamilla

**Affiliations:** Yale School of Public Health, New Haven, CT, United States

**Keywords:** food security, nutrition policy, dietary quality, measurement, nutrition security, food access

## Abstract

Food security (FS) is a powerful social determinant of health (SDOH) and is crucial for human and planetary health. The objectives of this article are to (i) provide clarity on the definitions of FS and nutrition security; (ii) provide a framework that clearly explains the links between the two constructs; (iii) summarize measurement approaches, and (iv) illustrate applications to monitoring and surveillance, policy and program design and evaluation, and research, mainly based on the ongoing rich experience with food insecurity (FI) scales. A clear and concise definition of FI and corresponding frameworks are available. There are different methods for directly or indirectly assessing FI. The best method(s) of choice need to be selected based on the questions asked, resources, and time frames available. Experience-based FI measures disseminated from the United States to the rest of the world in the early 2000s became a game changer for advancing FI research, policy, program evaluation, and governance. The success with experience FI scales is informing the dissemination, adaptation, and validation of water insecurity scales globally. The many lessons learned across countries on how to advance policy and program design and evaluation through improved FS conceptualization and measurement should be systematically shared through networks of researchers and practitioners.

## Introduction

Food security (FS) is a powerful social determinant of health (SDOH) and is crucial for human and planetary health (1). FS is indeed crucial for nations to meet the Sustainable Development Goals (SDGs) and, in turn, the SDGs need to be met to achieve food and nutrition security for all (1). Unfortunately, there are still many misunderstandings and misconceptions about the definition of the construct of FS, how it relates to nutrition security, and which sound frameworks are needed to guide the research and practice work in this field (2). Hence, the objectives of this article are to (i) provide clarity on the definitions of FS and nutrition security; (ii) provide a framework that clearly explains the links between the two constructs; (iii) summarize measurement approaches, and (iv) illustrate applications to monitoring and surveillance, policy and program design and evaluation, and research, mainly based on the ongoing rich experience with food insecurity (FI) scales.

Based on the 1996 World Summit in Rome hosted by the United Nations Food and Agriculture Organization (3), the United Nations World Food Security Committee defines FS as a condition that exists when “…people, at all times, have physical, social and economic access to sufficient, safe and nutritious food which meets their dietary needs and food preferences for an active and healthy life” (4).

Consistent with the United Nations World Food Security definition, the U.S. Department of Agriculture (USDA) has defined FS as “access by all people at all times to enough food for an active, healthy life” (5) and specified that “Food security includes at a minimum: (i) the ready availability of nutritionally adequate and safe foods, and (ii) the assured ability to acquire acceptable foods in socially acceptable ways (e.g., without resorting to emergency food supplies, scavenging, stealing, or other coping strategies),” hence endorsing the dimension of social acceptability as a core component of the FS construct (5). Furthermore, the US has expressed that an active, healthy life depends on both adequate amounts of food and the proper mix of nutrient-rich food to meet an individual’s nutrition and health needs (ERS-USDA). As a corollary, FI has been defined as a condition that occurs “whenever the availability of nutritionally adequate and safe foods, or the ability to acquire acceptable foods in socially acceptable ways is limited or uncertain” (5).

The definition of FS that has been in place for over three decades has made it clear that FS is a multidimensional construct that includes the following dimensions: *Quantity*, enough calories; Dietary *Quality*, nutritional value of foods; *Food Safety*, foods free of harmful microorganisms or other environmental contaminants; *Suitability*, culturally acceptable; *Psycho-emotional*, anxiety and feelings of deprivation; and *Social Acceptability*, socially acceptable methods for acquiring foods (1, 6, 7).

These definitions of FS were strongly informed by the development of FS experience-based scales based on mixed-methods research conducted with people in the US experiencing FI and hunger (the most extreme form of FI) (8), and led to the development, validation, and launch of the US Household Food Security Survey (USHFSSM) module in 1995 (5) and its subsequent dissemination, adaptation, and validation globally (7, 9, 10).

**Figure 1:**
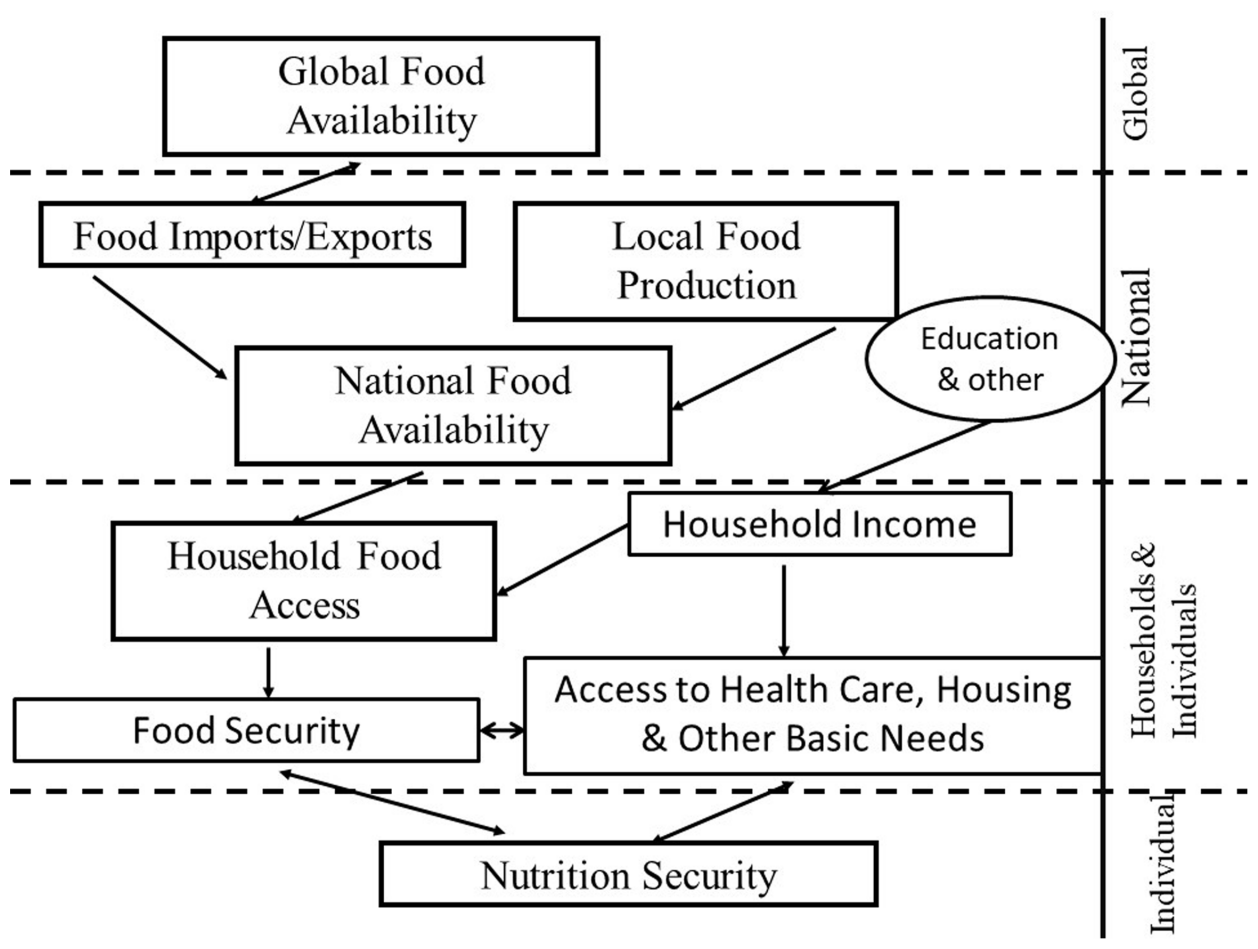
The relationships between global food security, household food security, and nutrition security. Adapted with permission from Smith (12), Frankenberger et al. (13), and UNICEF (14).

Nutrition status has been defined as “the assimilation and utilization of nutrients by the body plus interactions of environmental factors such as those that affect food consumption and food security” (11). Hence, it is a construct that needs to be assessed and understood by researchers, program evaluators, and policymakers at the level of the individual’s organism. Indeed, Smith presented a clear food and nutrition security multilevel framework (12) adapted from Frankenberger (13) and UNICEF (14), ranging from the global to the individual level to understand the strong relationship between FS and nutrition security and their distinct characteristics (Figure 1).

Extensive research involving experience-based FS scales has shown that in human societies, FS needs to be understood at the household level, and that it is a SDOH that, in turn, is strongly determined by socio-economic status and social class (7). FS relies on stable economic, physical, and social access to diverse, healthy, and nutritious foods that are culturally acceptable in the communities where the households are located. This access, in turn, depends on regional, national, and global availability of such foods. Currently, the global availability of these foods is constantly threatened by climate change and armed conflicts across the globe (1).

Nutrition security among individuals is determined by FS in combination with other SDOH, including healthcare access, housing, and other basic human needs such as water security (3, 4, 15).

Food and nutrition security sits right at the intersection of public health and human rights, as reflected in articles from the UN Charter on the Right to Adequate Food (16). For instance, *Articles 11 and 12* of the *International Covenant on Economic, Social, and Cultural Rights (ICESCR)* along with children’s rights to food, health, care, survival, and development; *Articles 6, 24, and 27 of the Convention on the Civil Rights of the Child (CRC)* detailing the rights of mothers to adequate nutrition during pregnancy and lactation; and Article *12.2 of the Convention on the Elimination of all forms of Discrimination Against Women (CEDAW)* highlight this intersection. These articles reflect the universal, indivisible, interrelated, and interdependence of the human right to food.

The definition of food and nutrition security in Brazil is an example of how a country can incorporate the domains of human rights, taking the SDOH and environmental sustainability into account. Specifically, the Brazilian Government defines food and nutrition security based on its Organic Law on Food and Nutritional Security (LOSAN—Law No. 11.346, issued on 15 September 2006) as “the realization of everyone’s right to regular and permanent access to quality food, in sufficient quantity, without compromising access to other essential needs, based on health-promoting food practices that respect cultural diversity and are environmentally, culturally, economically and socially sustainable” (17).

It is clear from the definitions of FS used internationally and within countries that the construct of FS has four interrelated dimensions: food availability, access, utilization, and stability (4). Since access to food is key for food and nutrition security, it is important to understand what this construct means and its domains. Food access centers on the stable availability of nourishing, affordable, and suitable food access, shaped by diverse economic, social, commercial, and political structural factors. Physical and economic access to nutritious foods coming from sustainable food production systems are important elements of the food access construct. Hence, the construct of food access has five dimensions: food availability, proximity, affordability, acceptability, and accommodation to cultural preferences (18).

Food and nutrition security can only be attained with stable access to healthy, nutritious, and sustainable diets. These diets should avoid or strongly minimize the inclusion of ultra-processed foods and beverages and maximize the intake of unprocessed or minimally processed foods such as fresh fruits and vegetables, whole grains, and sustainable protein sources, prepared in healthy ways, as well as water (19–21).

### Food security assessment methods

There are different methods to assess different dimensions of FS, including aggregated availability to adequate calories and FAO balance sheets; individual-level dietary intake with 24-h recalls, Food Frequency Questionnaires, and/or food records; anthropometry; and biomarkers such as blood levels of iron and other micronutrients (6). However, the only method currently available to directly assess household FS is through experience-based scales, almost all of which are derived from the USHFSSM (6, 22). All the methods have strengths and weaknesses related to specificity, ease of application, data collection speed, cost, and measurement errors, they complement each other, and the choice of method(s) depends on the question(s) being asked (22). For example, a comprehensive assessment of the nutritional status of individuals requires evaluation of their food consumption patterns and FI status as well as examining biochemical, clinical, and anthropometric indices of their nutritional status (11).

Given the rapid dissemination and utilization of experience-based scales globally, the following subsection focuses on them.

#### Experience-based food security scales

The origin of experience-based scales dates back to the 1980s when ethnographic research conducted in upstate New York with people who had experienced hunger and FI suggested that FI could be understood as a stepwise process that starts with household members worrying about food running out followed by sacrificing dietary quality and eventually calories are first reduced among adults and last among children living in the household (6). Subsequently, FS experience-based scales were developed by researchers to capture this sequence of events as reported by a household informant. The strong validity of the scale provided a strong impetus for the US Government to bring together a group of experts to develop what became the USHFSSM, which was heavily influenced by the Radimer/Cornell Hunger scale (23) and the Community Childhood Hunger Identification Project (CCHIP) scale (5, 24, 25). As a result, the USHFSSM has been incorporated since 1995 in the US Census Bureau Continuous Population Survey (CPS) (5) and became incorporated in nationally representative surveys such as the National Health and Nutrition Examination Survey (NHANES) (25) (Table 1).

**Table 1:** The US Household Food Security Survey Module.^a^^,^^b^

#	Question	Response options
**Items in reference to the whole household** ^c^		
1	The first statement is “(I/We) worried whether (my/our) food would run out before (I/we) got money to buy more.” Was that often true, sometimes true, or never true for (you/your household) in the last 12 months?	Often true Sometimes true Never true DK or Refused
2	The food that (I/we) bought just did not last, and (I/we) did not have money to get more. Was that often, sometimes, or never true for (you/your household) in the last 12 months?	Often true Sometimes true Never true DK or Refused
3	(I/we) could not afford to eat balanced meals. Was that often, sometimes, or never true for (you/your household) in the last 12 months?	Often true Sometimes true Never true DK or Refused
**Items in reference to adults in the household** In the last 12 months…		
4	Did (you/you or other adults in your household) ever cut the size of your meals or skip meals because there was not enough money for food?	
4a	How often did this happen—almost every month, some months but not every month, or in only 1 or 2 months?	Almost every month Some months but not every month Only 1 or 2 months DK
5	Did you ever eat less than you felt you should because there was not enough money for food?	Yes No DK
6	Were you ever hungry but did not eat because there was not enough money for food?	Yes No DK
7	Did you lose weight because there was not enough money for food?	Yes No DK
8	Did (you/you or other adults in your household) ever not eat for a whole day because there was not enough money for food?	Yes No DK
8a	How often did this happen—almost every month, some months but not every month, or in only 1 or 2 months?	Almost every month Some months but not every month Only 1 or 2 months DK
**Items in reference to children in the household** In the last 12 months…		
9	(I/we) relied on only a few kinds of low-cost food to feed (my/our) (child/the children) because (I was/we were) running out of money to buy food in the last 12 months?	Often true Sometimes true Never true DK or Refused
10	Could not feed (my/our) (child/the children) a balanced meal, because (I/we) could not afford that in the last 12 months?	Often true Sometimes true Never true DK or Refused
11	(My/Our child was/The children were) not eating enough because (I/we) just could not afford enough food in the last 12 months?	Often true Sometimes true Never true DK or Refused
12	Did you ever cut the size of (your child’s/any of the children’s) meals because there was not enough money for food?	Yes No DK
13	Did (CHILD’S NAME/any of the children) ever skip meals because there was not enough money for food?	Yes No DK
13a	How often did this happen—almost every month, some months but not every month, or in only 1 or 2 months?	Almost every month Some months but not every month Only 1 or 2 months DK
14	(Was your child/were the children) ever hungry but you just could not afford more food?	Yes No DK
15	Did (your child/any of the children) ever not eat for a whole day because there was not enough money for food?	Yes No DK

a Adapted from: https://www.ers.usda.gov/topics/food-nutrition-assistance/food-security-in-the-u-s/survey-tools/#household.

b Questions 1 through 15 (including 1a, 4a, and 13a) comprise the 18-item U.S. Household Food Security Scale (questions 1 through 8a for households with no child present). Specification of food security status depends on the raw score and whether there are children in the household (i.e., whether responses to child-referenced questions are included in the raw score).

c If single adult in the household, use “I,” “My,” and “You” in Parentheticals; otherwise, use “we,” “our,” and “your household”.

For households with one or more children: Raw score zero—High food security; 1-2—Marginal food security; 3-7—Low food security; 8-18—Very low food security. For households with no child present: Raw score zero—High food security; 1-2—Marginal food security; 3-5—Low food security 6-10—Very low food security. Households with high or marginal food security are classified as food secure. Those with low or very low food security are classified as food insecure. Questions 1 through 8a (including 4a) comprise the U.S. Adult Food Security Scale. Raw score zero—High food security among adults; 1-2—Marginal food security among adults; 3-5—Low food security among adults; 6-10—Very low food security among adults. Questions 2 through 6 (including 4a) comprise the six-item Short Module from which the six-item Food Security Scale can be calculated. Raw score 0-1—High or marginal food security (raw score 1 may be considered marginal food security, but a large proportion of households that would be measured as having marginal food security using the household or adult scale will have a raw score of zero on the six-item scale); 2-4—Low food security; 5-6—Very low food security. Questions 9 through 15 (including 13a) comprise the U.S. Children’s Food Security Scale. Raw score 0-1—High or marginal food security among children (raw score 1 may be considered marginal food security, but it is not certain that all households with a raw score of zero have high food security among children because the scale does not include an assessment of the anxiety component of food insecurity); 2-4—Low food security among children; 5-8—Very low food security among children.

The specific, measurable, achievable, relevant, and time-bound (SMART) properties of the indicators derived from the USHFSSM led to the global dissemination, adaptation, and validation of the USHFSSM across world regions (26). In Latin America, the experience of the Brazilian Food Insecurity Scale (EBIA) (27, 28), a scale from Colombia (9), and the Household Food Insecurity Access Scale (HFIAS) (29) led to the development of the Latin American Food Security Scale (ELCSA) in strong partnership with FAO’s Latin American regional office in Chile (9) (Table 2).

**Table 2:** Latin American and Caribbean Food Security Scale (ELCSA).^1^

**Questions referring to the household or adults living in the household** ^2^ *During the last 3 months, because of lack of money or other resources…*
1 Were you worried about running out of food?
2 Did your household run out of food at any time?
3 Was your household unable to eat a healthy and nutritious diet?
4 Did you or anybody else in your household usually have to eat the same kinds of foods almost every day?
5 Did any day, you or any other adult in your home skip breakfast, lunch, or dinner?
6 Did any adult in your home eat less food than what you think s/he needed because there was not enough food?
7 Was there any day when you or any other adult in your home felt hungry but did not eat because there was not enough food?
8 Was there any day when you or any other adult in your home did not eat for a whole day or just ate once during the day because there was not enough food during the last 3 months?
**Questions for households with minors under 18 years of age** ^**2**^ *During the last 3 months, because of lack of money or other resources…*
9 Did any children/youth in your household unable to consume a healthy and nutritious diet?
10 Did any children/youth in your household usually have to eat the same kinds of foods almost every day?
11 Did any child/youth in your household eat less food than what s/he needs because there was not enough food?
13 Did any day you have to reduce the amount of food served to children/youth in your household?
14 Was there any day when any child/youth in your household felt hungry but could not be fed because there was not enough food?
15 Was there any day when any child/youth in your household did not eat for a whole day or just ate once during the day because there was not enough food?

^1^Adapted from Comité Científico de la ELCSA (9). Escala Latinoamericana Y Caribeña De Seguridad Alimentaria (ELCSA): Manual De Uso y Aplicaciones. FAO, Santiago, Chile. Available from http://www.fao.org/3/i3065s/i3065s.pdf. ^2^Response options: Yes, No, Do not Know, Refuse. An additive score is computed based on the number of questions affirmed, Cutoff points for households with children/youth: ‘household food secure’ (score = 0), ‘mild household food insecurity (score = 1–5), ‘moderate household food insecurity’ (score = 6–10), ‘severe household food insecurity’ (score = 11–15). Cutt-off points for households with members above the age of 18: ‘household food secure’ (score = 0), ‘mild household food insecurity’ (score = 1–3), ‘moderate household food insecurity’ (score = 4–6), ‘severe household food insecurity’ (score = 7–8).

ELCSA was subsequently adopted in additional countries, including Mexico and Guatemala, and it eventually provided the impetus for the development of the Food Insecurity Experience Scale (FIES) that is being used by FAO to track the Sustainable Development Goal 2.1.2 (10) (Table 3).

**Table 3:** Food Insecurity Experience Scale.^a^^,^^b^

Question	Response options	
Q1. During the last 12 months^c^,**was there a time when you were worried you would not have enough food to eat because of a lack of money or other resources?**	0 No 1 Yes	98 DK 99 Refused
Q2. Still thinking about the last 12 months,**was there a time when you were unable to eat healthy and nutritious food because of a lack of money or other resources?**	0 No 1 Yes	98 DK 99 Refused
Q3. During the last 12 months,**was there a time when you ate only a few kinds of foods because of a lack of money or other resources?**	0 No 1 Yes	98 DK 99 Refused
Q4. During the last 12 months,**was there a time when you had to skip a meal because there was not enough money or other resources to get food?**	0 No 1 Yes	98 DK 99 Refused
Q5. Still thinking about the last 12 months,**was there a time when you ate less than you thought you should because of a lack of money or other resources?**	0 No 1 Yes	98 DK 99 Refused
Q6. In the past 12 months,**was there a time when your household ran out of food because of a lack of money or other resources?**	0 No 1 Yes	98 DK 99 Refused
Q7. In the past 12 months,**was there a time when you were hungry but did not eat because of a lack of money or other resources for food?**	0 No 1 Yes	98 DK 99 Refused
Q8. During the last 12 months,**was there a time when you went without eating for a whole day because of a lack of money or other resources?**	0 No 1 Yes	98 DK 99 Refused

a Adapted from https://www.fao.org/3/bl404e/bl404e.pdf.

b For household level (vs. individual level) FI assessment substitute ‘was there a time when you…you’ with ‘was there a time when you or others in your household…’ Raw additive scores are used to classify households into food secure, mildly FI, moderately FI, or severely FI.

c Time frame can also be the previous 4 weeks. In this case, if questions 6, 7, and 8 are affirmed, then each of them needs to be followed by ‘How often did this happen in the past 4 weeks?’ with response options: Rarely (1 or 2 times); Sometimes (3–10 times); Often (more than 10 times); Do not Know; Refused.

**Figure 2:**
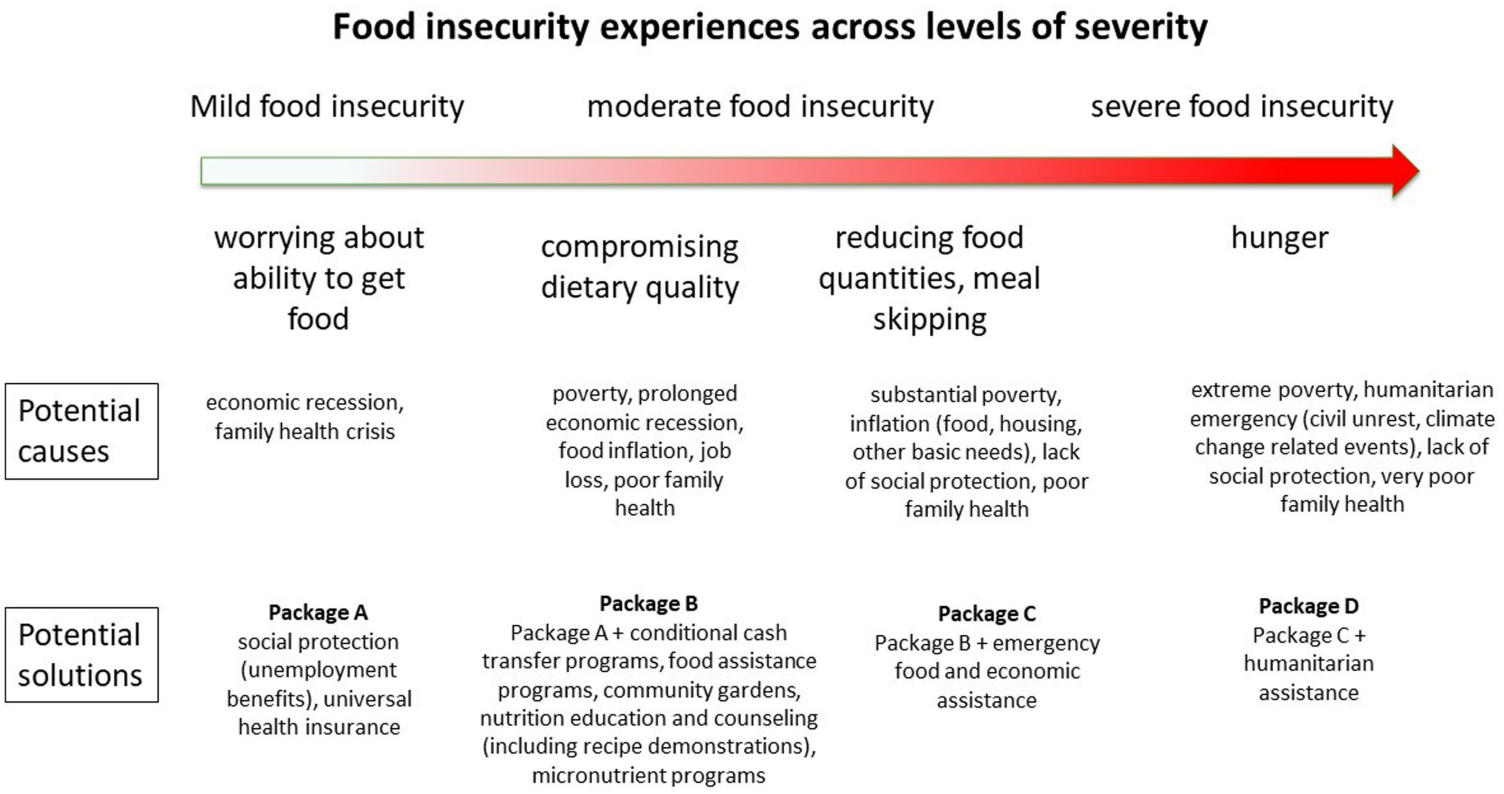
Food insecurity experiences across levels of severity. Potential causes and solutions. Prepared by the author.

FS experience scales yield an additive score that allows households to be classified according to their level of severity of FI (mild, moderate, and severe), which has allowed for a better understanding of how to design and target FS policies and programs (6). This is because different levels of severity of FI represent different issues ranging from psycho-emotional stress to poor dietary quality all the way to excessive hunger, which requires different solutions (30) (Figure 2).

### Application of food security experience scales across world regions

FS experience scales have allowed countries, regions, and the world to have better estimates of the burden of FI in the world. Based on FIES, in 2022, 29.6% of the global population, or 2.4 billion people, were moderately or severely FI (31). This meant that there were 391 million more people experiencing moderate or severe FI in 2022 than in 2019, before the outbreak of the COVID-19 pandemic (31). Furthermore, significant inequities existed based on the economic development of countries, the area of residence (rural vs. peri-urban vs. urban), and sex (female vs. male) (31).

FS experience scales have allowed researchers to better understand the links between FI and (i) the triple burden of malnutrition (undernutrition, obesity, and climate change) (32); (ii) infectious diseases including COVID-19, and common childhood communicable diseases in low- and middle-income countries; (iii) poor mental health across the life course; and (iv) poor early childhood development; (v) and poor medication adherence to treatments (1, 7, 30–39).

Furthermore, from a policy and programmatic perspective, FS experience scales have been useful for supporting equitable social policy investments (30, 31) across countries and for holding governments accountable when FI rates increase, as recently shown in Brazil, and the number of people affected by severe FI increased from 10 million to 30 million between 2018 and 2022 (40, 41). They have also been used to assess the impact of specific programs, including the SNAP programs in the US (26) and conditional cash transfer programs in Mexico (42) and Brazil (40).

### Food and water insecurity

The profound link between water and FI in a highly unstable world highlighted the need to consider the use of water insecurity experience scales such as the Household Water Insecurity Experiences (HWISE) alongside the FI scales (15). The 12-item HWISE scale yields an additive score that, combined with a pre-established cutoff point, allows households to be categorized as water-secure or insecure (43) (Table 4). HWISE assesses the frequency in the previous 4 weeks that anyone in the household experienced any of 12 negative emotions (e.g., worry, anger, and shame), disruptions in daily life (e.g., inability to wash clothes, hands, or take a bath), or even unsatisfied thirst due to water insecurity. Research using HWISE has shown that WI is strongly and consistently associated with FI all over the world (15, 44), as well as with physical and mental health outcomes (45–48). Similar to food, access to safe water is a human right recognized by the UN charter since 2010 (15, 43), and it is important to track it as part of the SDGs with an experience scale as it is done for FS (45).

**Table 4:** Household Water Insecurity Experience Scale (HWISE).^a^^,^^b^

Dimension	Question
Worry	1 In the last 4 weeks, how frequently did you or anyone in your household worry you would not have enough water for all of your household needs?
Interrupt	2 In the last 4 weeks, how frequently has your main water source been interrupted or limited (e.g., water pressure, less water than expected, and river dried up)?
Clothes	3 In the last 4 weeks, how frequently have problems with water meant that clothes could not be washed?
Plans	4 In the last 4 weeks, how frequently have you or anyone in your household had to change schedules or plans due to problems with your water situation? (Activities that may have been interrupted include caring for others, doing household chores, agricultural work, income-generating activities, sleeping, etc.)
Food	5 In the last 4 weeks, how frequently have you or anyone in your household had to change what was being eaten because there were problems with water (e.g., for washing foods, cooking, etc.)?
Hands	6 In the last 4 weeks, how frequently have you or anyone in your household had to go without washing hands after dirty activities (e.g., defecating or changing diapers, cleaning animal dung) because of problems with water?
Body	7 In the last 4 weeks, how frequently have you or anyone in your household had to go without washing their body because of problems with water (e.g., not enough water, dirty, unsafe)?
Drink	8 In the last 4 weeks, how frequently has there not been as much water to drink as you would like for you or anyone in your household?
Angry	9 In the last 4 weeks, how frequently did you or anyone in your household feel angry about your water situation?
Sleep	10 In the last 4 weeks, how frequently have you or anyone in your household gone to sleep thirsty because there wasn’t any water to drink?
None	11 In the last 4 weeks, how frequently has there been no useable or drinkable water whatsoever in your household?
Shame	12 In the last 4 weeks, how frequently have problems with water caused you or anyone in your household to feel ashamed/excluded/stigmatized?

a Adapted from https://arch.library.northwestern.edu/concern/generic_works/kk91fk74c.

b Each item is phrased to capture experiences that anyone in the household has had in the last 4 weeks. Responses to items are never (0 times), rarely (1–2 times), sometimes (3–10 times), often (11–20 times), and always (more than 20 times). Never is scored as 0, rarely is scored as 1, sometimes is scored as 2, and often/always is scored as 3. Households with a score > 12 are classified as water insecure.

Inspired by the experience with EBIA, Brazil recently applied the HWISE in a nationally representative sample to document the prevalence of WI during the COVID-19 pandemic and how strongly it relates to FI (41). Findings showed that 12% of households experienced WI in Brazil and that among those with WI, 42% experienced severe FI (vs. 12.1% in water-secure households) (43). Mexico has now also included HWISE and a water intermittency scale in nationally representative surveys. The application of HWISE in the National Health and Nutrition Survey (ENSANUT)-2021 demonstrated that HWISE has strong psychometric and predictive validity in the Mexican context (49), and its application through another nationally representative public opinion poll showed that 32% of Mexican households experienced water insecurity and that 68% of households experiencing severe FI were also experiencing WI (vs. 17% in FS households) (50). Furthermore, the application of a water intermittency scale in ENSANUT-2022 found that only 31.5% of Mexican households had water 7 days per week, and of these, only 17.4% did not experience water scarcity in the previous 12 months (51). As expected, water intermittency was more common in the poorest region of Mexico and among the poorest families, confirming that the distribution of WI follows the same social, economic, and demographic inequity patterns as FI.

### Cross-border lessons learned

There are indeed key lessons learned that show how cross-border collaborations have advanced and can continue advancing FI solutions across borders and world regions.

The strong global consensus on the definition of FI and the development of sound conceptual frameworks explaining its determinants at multiple levels and how, together with other SDOH links with nutrition security, allowed for the development of FI measurement approaches that have helped understand the causes, consequences, and potential solution to FI across and within countries (1, 7, 9, 10, 26).

The capacity of countries, regions, and the world to track FI with SMART monitoring and surveillance systems on a continuous basis has been greatly facilitated by the dissemination, adaptation, and validation of the USHFSSM (1, 7, 9, 10, 26). In the US, this scale has been used through the CPS Food Security Supplement, NHANES, the National Health Interview Survey (NHIS), the Early Childhood Longitudinal Survey (ECLS), and other monitoring and surveillance systems across sectors. Latin American countries have included scales derived from the USHFSSM, such as EBIA (28) and ELCSA (9), as part of the countries’ national health and nutrition surveys, household income expenditure surveys, public opinion polls, and state and local monitoring systems. At a global level, FIES is used to track SDG 2.1.2, and in fact, FIES was instrumental in the addition of this target to the SDGs. As previously mentioned in this article, all the methods for assessing FI complement each other. Hence, it is encouraging that comprehensive multi-methods monitoring systems have also been developed, such as the food systems dashboard (52) and a low-burden tool for collecting valid, comparable food group consumption data through the “What the World Eats” initiative (53, 54).

FI experience scales have been shown to be helpful for food and nutrition security policies and program designs, including program targeting and evaluation. A robust body of evidence confirms that FI experience scales yield SMART indicators that can help improve FS governance across countries and regions (17, 26, 40, 55).

## Conclusion

A clear and concise global definition of FI and corresponding frameworks are in place. Countries such as Brazil have strengthened the definition of food and nutrition security by incorporating human rights and the sustainability dimension, which they have clearly operationalized through the country’s progressive food and nutrition security policies and dietary guidelines (21). There are different methods for directly or indirectly assessing FI. The best method(s) of choice need to be selected based on questions asked, resources, and time frames available. Experience-based FI measures disseminated from the United States to the rest of the world in the early 2000s became a game changer for advancing FI research, policy, and program evaluation. The success of experience-based FI scales is informing the dissemination, adaptation, and validation of WI scales globally. The rich lessons learned across countries on how to advance policy and program design and evaluation through improved FS conceptualization and measurement should be systematically shared through networks of researchers and practitioners such as the recently established Water Insecurity Experiences-Latin America and the Caribbean (WISE-LAC) Network (56).

## Author contributions

RP-E: Conceptualization, Investigation, Project administration, Writing – original draft, Writing – review & editing.
